# Superficial Keratectomy: A Review of Literature

**DOI:** 10.3389/fmed.2022.915284

**Published:** 2022-07-06

**Authors:** Farhad Salari, Alireza Beikmarzehei, George Liu, Mehran Zarei-Ghanavati, Christopher Liu

**Affiliations:** ^1^Eye Research Center, Farabi Eye Hospital, Tehran University of Medical Sciences, Tehran, Iran; ^2^Medical School, Tehran University of Medical Sciences, Tehran, Iran; ^3^School of Medicine, Anglia Ruskin University, Chelmsford, United Kingdom; ^4^Tongdean Eye Clinic, Brighton, United Kingdom; ^5^Sussex Eye Hospital, Brighton, United Kingdom; ^6^Brighton and Sussex Medical School, Brighton, United Kingdom

**Keywords:** superficial keratectomy, diamond burr polishing, epithelial debridement, manual keratectomy, mitomycin C

## Abstract

Superficial keratectomy (SK) is the manual dissection of the superficial corneal layers (epithelium, Bowman's layer, and sometimes superficial stroma). SK is done using a surgical blade or diamond burr. Some surgeons use intraoperative mitomycin C 0.02% or amniotic membrane transplantation to improve surgical outcomes. This literature review shows that SK remains an effective method for different indications, including tissue diagnosis, excision of corneal degenerations, dystrophies, scarring, recurrent corneal erosions, and retained corneal foreign body.

## Introduction

Superficial keratectomy (SK) was one of the first methods to treat corneal opacity and diagnose corneal lesions after its introduction in 1952 (1). SK is defined as the manual dissection of superficial corneal layers (epithelium, Bowman's layer, and sometimes superficial stroma) without tissue replacement (2). Superficial keratectomy, epithelial basement debridement and epithelial debridement are often incorrectly interchangeably used in literature. Epithelial debridement is the surgical excision of the epithelium without the scraping of the basement membrane with a sponge or blade. Alcohol delamination is a type of epithelial debridement in which the loosening of epithelium is done using alcohol. Epithelial basement debridement is the surgical removal of the epithelium and the scraping of the epithelial basement membrane using a blade or sponge (3).

In this study, we consider epithelial basement membrane debridement as a type of superficial keratectomy. Despite excimer laser associated techniques like phototherapeutic keratectomy (PTK) being introduced over the past decades for treatment and evaluation of corneal lesions, SK still remains a powerful method for various indications, including tissue diagnosis, excision of corneal degenerations, dystrophies, scarring, recurrent corneal erosions and retained corneal foreign body. Herein, we narratively review the literature to illustrate indications, surgical techniques, and outcomes of superficial keratectomy.

## Methods

We searched Pubmed, Embase, and Medline databases using “superficial keratectomy”, “manual keratectomy”, “epithelial debridement”, and “diamond burr polishing” in July 2021. In addition, we reviewed references from extracted papers. After deduplication, two reviewers (FS, SM) independently screened the titles and abstracts (Appendix 1).

**Figure 1:**
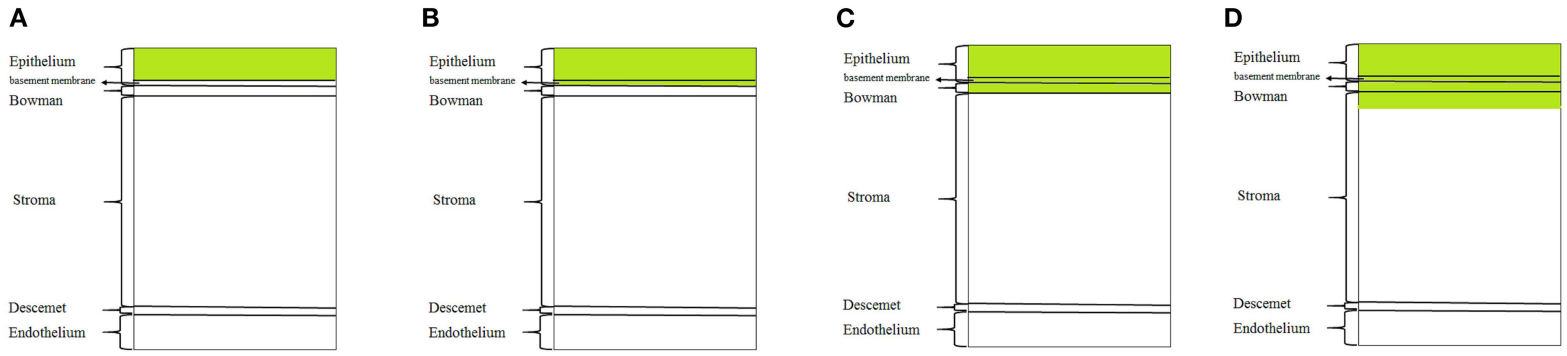
Schematic portraying the region of corneal tissue excised for various keratectomy techniques. **(A)** epithelial debridement or simple alcohol delamination; **(B)** epithelial basement membrane debridement; **(C,D)** Superficial keratectomy with diamond burr polishing or blade.

All studies that reported technique, indication, and outcomes of superficial keratectomy were included. In this study, we consider epithelial basement membrane debridement as a type of superficial keratectomy, but we don't consider alcohol delamination and epithelial debridement as a subtype of SK (Figure 1). Some studies used superficial keratectomy and epithelial debridement interchangeably. We decided to contain or exclude them based on the reported method of surgery. Studies that only evaluated phototherapeutic keratectomy (PTK) were excluded.

## Surgical Technique And Postoperative Management

SK is a safe procedure as none of the reports and series in the literature reported intraoperative complications. However, patients should be informed that discomfort due to epithelial debridement might persist for several days. There are some contraindications for SK. Although in SK, we remove superficial corneal layers, there is a small risk of corneal haze. Thus, for indications like bullous keratopathy in patients with a good visual prognosis, SK without endothelial keratoplasty may be considered a relative contraindication. SK in patients with ocular surface diseases like severe dry eye syndrome or lid problems that endanger normal healing of epithelium may also persuade surgeons to choose a substitute method or postpone the operation after optimization of the ocular surface.

The surgeon may choose topical, peri, or retrobulbar anesthesia based on the level of cooperation. The procedure usually is done by a microscope in the operating theater. Performing SK at the slit lamp is also possible but depends on patients' cooperation and the type of technique surgeons choose. The eye is prepared with povidone-iodine, and a lid speculum is inserted. Then, the superficial corneal layers are debrided with a sterile sponge or surgical blade. Some authors apply 20% alcohol for 40 s to debride the epithelium before scraping the cornea with a blade or sponge. Sayegh et al. suggested using cocaine 4% for 3–5 min to loosen the epithelium. They hypothesized the anesthetic effect of cocaine lessens the discomfort during the procedure (4). Furthermore, some surgeons remove the loose epithelium and then polish Bowman's layer with a diamond burr. Manual or automated diamond burr polishing helps debride persistent abnormalities. Additionally, there is another study that reports the application of Amoils epithelial scrubber to polish Bowman's layer (5). The following steps differ regarding the etiology that necessitated SK. Forceps may be utilized to grasp the nodule's edge and raise this edge firmly, to remove nodules, such as in Salzmann nodular degeneration (SND). If there is dystrophic tissue, the lesion is peeled off from Bowman's layer using a blunt blade or cellulose microsponge in one continuous plane. Sharp dissection may be performed if it is not possible to shave off the peripheral membrane from the limbus or if the surgeon finds making a smooth surface under the lesion is not possible without using sharp dissection (5, 6).

The surgeon may choose some adjuvant treatments based on the indication for SK. For example, in patients with band keratopathy, applying Ethylene diamine tetra-acetic acid (EDTA) helps dissolve calcific deposits (7). Another adjunctive medication is the use of a sponge soaked with mitomycin C 0.02% (MMC). MMC is an antimetabolite that decreases activated fibrocytes and keratocytes. It may subsequently prevent the recurrence of pathologies such as Salzmann nodular degeneration and lessens the chance of corneal haze following SK (8). Although MMC is toxic to the cornea, Teus et al. reported 0.02% MMC for <2 min of exposure time was not toxic (9).

After the procedure, a therapeutic contact lens should be placed, and the patient discharged with artificial tears, topical antibiotics and cycloplegic drops. Some ophthalmologists prescribe corticosteroid eye drops to suppress inflammation. In addition, topical non-steroidal anti-inflammatory drugs like ketorolac may alleviate pain. Patients are revisited in the first week postoperatively to ensure healing of the epithelial defect. After healing of the epithelial defects is confirmed, topical antibiotics are ceased, and topical steroids can be tapered.

## Indications For Superficial Keratectomy

SK is an available and effective method for various indications, including tissue diagnosis, excision of corneal degenerations, dystrophies, scarring, recurrent corneal erosions and retained corneal foreign body (Table 1). This part briefly explains the indication and outcomes, complications, and adjunct treatments for each indication separately.

**Table 1:** Indications for superficial keratectomy.

**Common**	**Rare**
Band Keratopathy	Fuchs' Superficial Marginal Keratitis infectious crystalline keratopathy corneal haze after PRK
Keratitis	Corneal Neovascularization diagnosis of “Contact Lens-Related Keratoschisis”
Pterygium excision	Climatic droplet keratopathy
Amniotic membrane transplantation	Reis Buckler dystrophy
Squamous cell carcinoma of the cornea	Superficial Juvenile Granular Dystrophy map-dot-fingerprint basement membrane dystrophy
Anterior basement membrane dystrophy peripheral hypertrophic subepithelial corneal degeneration	Neurotrophic epithelial defect
Corneal foreign body removal	Mooren's ulcer
Bullous keratopathy	Secondary Gelatinous-Like Keratopathy
Salzmann's nodular degeneration	Corneal keloid
Recurrent epithelial erosion	Posterior polymorphous corneal dystrophy

### Recurrent Corneal Erosion Syndrome

Recurrent corneal erosion syndrome (RCES) is a relapsing breakdown of the corneal epithelium and the Bowman's layer. Trauma and corneal dystrophies are considered the main etiology (10). Many patients respond to conservative therapy with a combination of lubrication with drops or gels and bandage contact lenses. However, some people experience symptoms that are refractory to conservative topical therapy and require surgery. Many treatment options are available for the treatment of RCES, but there is no consensus on which is the best. Options include epithelial debridement (ED), SK, anterior stromal puncture (ASP), Nd: YAG laser treatment, alcohol delamination, and PTK (11). SK is one of the most common interventions in patients with RCES. SK is a procedure that can be easily performed in an office, imposes a lesser cost to the healthcare system, and requires a lower skill level than PTK (5). A Cochrane review on interventions for RCES showed that performing diamond burr polishing in addition to the epithelial debridement decreases the recurrence significantly. Moreover, PTK may have a higher recurrence rate (373 per 1,000 vs. 294 per 1,000 in mean 24 months follow up) and lower symptom relief (378 per 1,000 vs. 590 per 1,000 in 3 months of follow up) in comparison with epithelial debridement (11).

The literature review revealed that most authors prefer SK with diamond burr polishing over simple SK. The recurrence rate ranged from 0 to 24% (Table 2). Most studies reported that best-corrected visual acuity (BCVA) was unchanged after SK. Unfortunately, there are sparse data regarding the change in corneal and refractive astigmatism after SK due to RCES. Wong et al. conducted a randomized trial to compare epithelial debridement (ED) with diamond burr superficial keratectomy (DBSK). They reported a significant reduction of astigmatism for the DBSK group but not for the ED group. They showed fewer major and minor recurrences and less need for reoperation in the DBSK group compared with the ED group; hence they concluded DBSK is a better option for RCES. Additionally, Sayegh et al. reported a reduction in the corneal surface irregularity after simple SK in epithelial basement membrane dystrophy patients (4). However, Yoo and Choi described a case of induced astigmatism after treating a 54-year-old man with DBSK (47). Another prevalent complication after SK is the subepithelial corneal haze. The incidence of corneal haze ranged from 14.3 to 26% (Table 2). Nonetheless, subepithelial corneal haziness mostly appears early postoperatively and does not persist (19). Activation of herpetic keratitis and corneal infiltration are rare complications of SK. In a series reported by Suri et al., the chance of corneal haziness was lower in the SK group compared to PTK (15).

**Table 2:** Outcomes of superficial keratectomy for different indications.

**Authors**	**Year of publication**	**Indication**	**Additional Techniques**	**Eyes: N**	**Follow up: mean (range in months)**	**Changes in BCVA**	**Changes in RA**	**Changes in CA**	**Recurrence: N (%)**	**Complications**
Bae et al. (12)	2018	RCES	ASP in 11 patients	21	6.5	20/36 to 20/32 (*p =* 0.29)	N/A	N/A	24	N/A
Vo et al. (13)	2015	RCES	DBSK	55	25.2	N/A	N/A	N/A	4	14.3% (Subepithelial haze)
Rac et al. (14)	2015	RCES	DBSK	8	18	N/A	N/A	N/A	0	N/A
Sayegh et al. (4)	2013	RCES	Topical cocaine	17	60	20/45 to 20/38 (P = 0.5)	N/A	SRI = 1.35 ± 0.42 to 0.79 ± 0.48	11	35% (Subepithelial haze)
Suri et al. (15)	2013	RCES	DBSK	68	23 (7-53)	N/A	N/A	N/A	14.8	22.2% (Subepithelial haze) 1% (Corneal infiltration)
Ryan et al. (16)	2013	RCES	DBSK	35	17.7	N/A	N/A	N/A	11	0
Reidy et al. (17)	2010	RCES	DBSK	4	N/A	N/A	N/A	N/A	N/A	N/A
Aldave et al. (18)	2009	RCES	DBSK	25	18.9	N/A	N/A	N/A	11	26% (Subepithelial haze) 1% (HSV keratitis) 1% (Corneal irregularity)
Wong et al. (19)	2009	RCES	DBSK	25	6	0.84 to 0.95 (P > 0.05)	0.70 to 0.44 (P = 0.04)	N/A	4 majors 20 minors	26% (Mild subepithelial haze) 4% (moderate subepithelial persistent haze)
Hodkin and Jackson (5)	2004	RCES	Amoils epithelial scrubber	26	21.2	20/43 to20/29 (p <0.05)	N/A	N/A	12	0
Sridhar et al. (20)	2002	RCES	DBSK in 1 ASP in 1 patient	27	6.7 ± 1	3 Better 17 unchanged 1 Worsen	N/A	N/A	11.	25.9% (subepithelial haze)
Soong et al. (21)	2002	RCES	DBSK	54	>3	20/26 to 20/22 (*p =* 0.002)	N/A	N/A	6	20%
He at al. (22)	2019	SND	-	3	N/A	Unchanged	2.7 ± 0.7 to 0.7 ± 0.5	6.1 ± 2.1 to 0.3 ± 0.9	0	N/A
Bae et al. (12)	2018	SND	-	42	4.6 ± 7 (1–31.75)	20/54 to 20/59 (*p =* 0.22)	3.99 to 2.82 D (*p =* 0.050)	2.95 to 2.65 D (*p =* 0.37)	0	N/A
Khaireddin et al. (23)	2011	SND	PTK + MMC	8	23.13 (12-31)	20/80 to 20/32 (*p <* 0.001)	SE change= +1.906 to −2.41	N/A	N/A	N/A
Graue-Hernández et al. (24)	2010	SND	-	41	61.2 (0–357)	20/37 to 20/34 (*p =* 0.57)	2.1 D (post-op N/A)	N/A	22	N/A
Malta et al. (25)	2008	SND	DBSK	4	(3-11)	3 Improved 1 Unchanged	N/A	N/A	0	No
Brown et al. (26)	2003	SND	MMC	30	28 (0.13–50)	20/100 to 20/40	2.33 to 1.94 D	N/A	0	N/A
Bae et al. (12)	2018	BK	EDTA	12	13.2 ± 15.7	20/159 to 20/107	N/A	N/A	17	No
Bee et al. (27)	2018	BK	DBSK	7	N/A	1 improved 3 unchanged 3 worsened	N/A	N/A	28	No
McGrath et al. (28)	2015	BK	MMC	16	17.8 ± 14.4	Log MAR 1.5 ± 1.5 to 1.4 ± 1.6	N/A	N/A	12.5	No
Rao et al. (29)	2008	BK	AMT in 9 EDTA in 2 patients	9	26.8 ± 10.2	6 improved 1 worsened 2 unchanged	N/A	N/A	0	2 scarring 1 retraction 1 graft loss, 1 ED
Anderson et al. (30)	2001	BK	AMT in 16 EDTA in 14	16	14.6 ± 2.9	4 improved 12 unchanged	N/A	N/A	25	1 exposed suture 1 exposure keratitis
Riedl et al. (31)	2021	PHSD	MMC	15	3	Log MAR 0.4 ± 0.2 to 0.21 ± 0.3 (*P <* 0.01)		Anterior corneal: 4.67 ± 2.4 to 1.4 ± 0.4 Posterior corneal: 0.6 ± 0.5 to 0.3 ± 0.2 (After 3 months)	0	No
McGrath et al. (28)	2015	Reis Buckler	MMC	3	N/A	N/A	N/A	N/A	N/A	N/A
Jarventausta et al. (32)	2014	PHSD	-	4	1	2 Improved 2 Worsened	3 Reduced 1 Unchanged	All Reduced	0	N/A
Gore et al. (33)	2013	PHSD	MMC	15	60(12- 132)	N/A	N/A	N/A	20	N/A
Rao et al. (29)			AMT	15	26.8 ± 10.2	12 improved 3 unchanged	N/A	N/A	6	20% amniotic membrane graft retraction
Maust et al. (34)	2003	PHSD	MMC	2	Case1: 8 Case 2: 15	N/A	N/A	N/A	0	N/A
Sajjadi et al. (35)	1992	Superficial Juvenile Granular Dystrophy	-	16	36	20/400 to 20/40	N/A	N/A	25	N/A
Bakhtiari et al. (36)	2013	Corneal Keloid	-	2	12 to 18	1 improved 1 unchanged	N/A	N/A	50	
Gupta et al. (37)	2016	Corneal Keloid	-	3	4 to 8	3 improved 1 unchanged	N/A	N/A	No	No
Lee et al. (38)	2016	Corneal Keloid	MMC in 2 AMT in 3	5	4 to 10	4 improved	N/A	N/A	100	No
McGrath et al. (28)	2015	Corneal scarring	MMC in 8 patients	10	16.8 ± 12.5	0.2 ± 0.2 to 0.1 ± 0.2	N/A	N/A	10	No
Ozgurhan et al. (39)	2013	Corneal scarring	DBSK+MMC	4	6	All improved	7 ± 0.8 to 1.4 ± 0.24	7.15 ± 2.82 to 1.4 ± 0.46	N/A	No
Khakshoor et al. (40)	2011	corneal haze after PRK	MMC	15	12	20/80 to 20/20 (*p <* 0.05)	N/A	N/A	0%	No
Qian et al. (41)	2008	Corneal neovascularization	subconjunctival Bevacizumab	3	N/A	unchanged	N/A	N/A	N/A	No
Bae et al. (12)	2018	EBMD	ASP in 11 patients	83	5.63 ± 7	20/47 to 20/40 (*p =* 0.033)	1.76 to1.15D (p < 0.010)	1.44 to1.06D (p < 0.022)	0	No
Vo et al. (14)	2015	EBMD	DBSK	36	33.2 (3.5–137.6)	20/33 to 20/29 (p < 0.002)	range−2.50 to1.75D (Median change 0.0D)	3.6 to1.8D (p < 0.001)	24	9.4% (subepithelial haze) 0.3% (HSV keratitis) 2% (marginal keratitis)
Sayegh et al. (4)	2013	EBMD	Topical cocaine	16	60 (1.3-96)	20/63 to 20/32 (*p <* 0.01)	N/A	SRI=1.47 ± 0.35 to 0.50 ± 0.30 (p < 0.001)	6	6% (subepithelial haze)
Aldave et al. (18)	2009	EBMD	DBSK	14	18.9 (3.5–66.5)	20/30 to 20/25 (*p =* 0.016)	N/A	N/A	2	N/A
Malta et al. (25)	2008	EBMD	DBSK	19	10.6 (3 o 39)	6 Unchanged 12 Improved 1 Worsened	N/A	N/A	0	5.2% (Subepithelial haze)
Itty et al. (42)	2007	EBMD	ASP in 13 patients	74	33	20/44 to 20/33 (*P <* 0.001)	1.2 to 1.1D (P = 1.0)	N/A	24	26% (subepithelial haze)
Tzelikis et al. (43)	2005	EBMD	DBSK	13	21.8	20/50 to 20/23 (p < 0.001)	N/A	N/A	0	No
Sridhar et al. (20)	2002	EBMD	DBSK ASP in 1 patient	27	6.7 ± 1	3 improved 17 unchanged 1 Worsened	N/A	N/A	11.1	25.9% (Subepithelial haze)
Buxton et al. (44)	1983	EBMD	DBSK	33	47	N/A	N/A	N/A	0	9% (Subepithelial haze)
Bae et al. (12)	2018	Bullous Keratopathy	ASP in case1 Gunderson flap in case2	2	N/A	unchanged	N/A	N/A	N/A	No
Shalabi et al. (45)	2014	Bullous Keratopathy	AMT	4	16	N/A	N/A	N/A	25	No
Fernandes et al. (46)	2011	Bullous Keratopathy	AMT	3	N/A	N/A	N/A	N/A	N/A	No

*AMT, amniotic membrane transplantation; ASP, Anterior stromal puncture; BCVA, best-corrected visual acuity; CA, corneal astigmatism; CDK, Climatic droplet keratopathy; DBSK, diamond burr superficial keratectomy; EBMD, Epithelial basement membrane dystrophy; ED, epithelial defect; EDTA, Ethylene diamine tetra-acetic acid; MMC, mitomycin C; N/A, not available; PHSD, Peripheral hypertrophic subepithelial degeneration; PRK, Photorefractive keratectomy; PTK, phototherapeutic keratectomy; RC, refractive astigmatism; RCES, Recurrent corneal erosion syndrome; SE: Spherical Equivalent; SND, Salzmann nodular degeneration; SRI, surface regularity index*.

SK effectively ameliorates ocular pain due to RCES. Current evidence suggests both simple SK and DBSK are safe and don't induce significant astigmatism. It seems DBSK is superior to ED for patients with RCES regarding its effectiveness in the reduction of corneal irregularity. However, there is no study directly comparing DBSK with SK. Considering its safety, availability, effectiveness, and skill level requirement, SK should be regarded as an effective treatment modality for treating RCES.

Epithelial basement membrane dystrophy (EBMD) is the most common anterior corneal dystrophy in which the basement membrane extends to the epithelium (map-dot-fingerprint appearance on slit lamp). This dystrophy is frequently reported as a reason for RCES or vision reduction, both leading to surgical interventions. The two main available interventions for EBMD are SK and PTK. Despite the prevalence of this dystrophy, there are sparse high-quality studies to compare these two surgical procedures. Sirdhar et al. performed a case-control study to compare results of PTK and SK with or without concomitant diamond burr polishing in patients with EBMD. After seven months of follow-up, there was no statistically significant difference in recurrence of symptoms or changes in BCVA among the two groups (20).

The review of papers on SK for EBMD showed that most of the studies reported BCVA significantly improved after SK. However, postoperative subepithelial haziness is not infrequent following SK for EBMD. Incidences of haziness ranged from 0 to 26% (Table 2). As SK had similar efficiency but at a lower cost than PTK, it should be considered the treatment of choice for clinically significant EBMD.

### Salzmann Nodular Degeneration

Salzmann nodular degeneration (SND) is a slowly progressive degenerative condition that is characterized by the appearance of nodular corneal opacity. It is a multifactorial disease that predominantly affects elderly females (48). The etiology of SND is mostly idiopathic. Other causes include keratitis, vernal keratoconjunctivitis (VKC), meibomian gland dysfunction, dry eye, trauma, measles and LASIK. Most cases with SND were successfully managed with lubrication and lid hygiene. A small number of patients require surgical interventions due to refractory symptoms or involvement of the visual axis. Surgical methods that can be undertaken are SK, PTK and SK plus PTK (26). There are reports of managing SND of various etiologies with SK: thyroid eye disease (49), meibomian gland dysfunction (50), and post LASIK (22, 51). Many modifications of SK have been proposed to reduce undesirable outcomes such as corneal haziness or reoccurrence of the symptoms. These include the application of MMC, adjunctive amnion membrane transplantation (AMT), and the use of adjunctive PTK. There are 6 case series treating SND with SK in the literature (Table 2). Only one series (8) reported enhancement of BCVA following SK. All other studies reported that BCVA did not significantly change postoperatively. All the studies that reported changes in refractive astigmatism reported reduced astigmatism postoperatively (Table 2). Recurrence was uncommon after SK for treating SND as new additional techniques such as MMC have been developed.

AMT in company with SK may ameliorate the healing process and minimize scarring. AMT is indicated when there is corneal thinning associated with SND. Multiple procedures may be required in recurrent cases of SND. Yoon et al. reported AMT after SK in a 75-year-old woman with recurrent episodes of SND (52). The patient underwent SK with AMT three times due to relapse of the disease. Furthermore, Rao et al. reported a case of using AMT after SK, which resulted in vision improvement (29). MMC has been proposed as an adjunctive treatment in SND. Various mechanisms have been suggested to explain why the application of MMC lowers the recurrence rate. The epithelial nature of nodules is more metabolically active than normal epithelial cells. It may be a key to understanding the effect of MMC as an antiproliferative drug (23). Bowers et al. reported the first series on intraoperative MMC. They applied a sponge soaked in MMC for 10 s in a series of 30 eyes with SND. They reported no recurrence in any of their cases. Some ophthalmologists also perform a combination of SK, PTK, and MMC to decrease the recurrence rate. Macron et al. reported a 91-year-old woman treated with SK, PTK, and MMC, which resulted in an improvement of BCVA (53). In addition, Khaireddin et al. reported data of surgery with the same method on eight eyes. SK+PTK+MMC resulted in myopic shift (reduction in hyperopic progression) and improvement of BCVA.

SK for treatment of SND significantly decreases symptoms, and with the aid of additional procedures, the recurrence rate is low. Additionally, this method has advantages over PTK as it is an easier method and is more cost-effective. Whereas SK is usually considered a first-line surgical option for SND, the surgeon should choose the best surgical method individually for each case with respect to the etiology, concurrent disease and previous treatments.

### Band Keratopathy

Band keratopathy (BK) is a corneal degeneration characterized by the deposition of calcium in subepithelial layers of the cornea. Surgical interventions for BK are SK, PTK, EDTA chelation, and AMT. Surgical interventions are indicated when calcium deposits endanger vision or induce ocular discomfort that is resistant to medical treatment. Debridement of calcific lesions is still the modality of choice despite the emergence of laser-based techniques. This is because PTK does not seem to offer better outcomes than SK, and there is an associated higher machine cost for PTK.

Our review of the literature showed that the reappearance after SK ranged from 12.5 to 28%. Postoperative complications were rare, although there were few cases of corneal scarring after SK (29). In SK, calcific lesions are manually excised from the cornea. Authors suggest additional techniques to enhance outcomes: EDTA chelation or AMT (Table 2). Esquenazi et al. reported a case of a 91-year-old man with recurrent BK treated with SK plus AMT to diminish pain and accelerate wound healing (54). In addition, Anderson et al. presented a case series treated with SK plus AMT. They show wound healing is accelerated with this method (30). Additionally, EDTA was shown to be efficient with minimal chance of intraoperative complications (29, 30). The postoperative recurrence rate in the BK series treated with EDTA ranged from 0 to 17%. Bee et al. reported a series of 9 eyes with limited visual potential with DBSK without using EDTA. The recurrence rate in these patients was 28% (27). Although there was no study directly comparing SK with and without the application of EDTA in the literature, it seems to be a useful adjunct as it could reduce the recurrence rate (20).

### Dystrophies and Degenerations

Peripheral hypertrophic subepithelial corneal degeneration (PHSCD) is a rare bilateral peripheral opacification of the cornea. This condition may create corneal irritation or induce refractive error, which may necessitate surgical intervention. PHSCD may cause progressive flattening of the central cornea, which results in significant astigmatism. Gore et al. reported that 7 of the 22 patients with PHSCD required surgical intervention (33). PTK, SK or SK+PTK may be planned. Jeng et al. reported a case of successfully reversing induced hyperopic and astigmatic shift in refraction by SK (55). The recurrence rate after SK ranged from 0 to 20 % (Table 2).

Superficial juvenile granular dystrophy is another rare dystrophy that may necessitate SK. Sajjadi et al. described a series of 16 eyes managed with SK. In the three-year postoperative follow-up period, 25% of cases presented with recurring symptoms. The postoperative BCVA was improved compared to the preoperative evaluation (35).

Reis-Bucklers' dystrophy is a disorder that involves Bowman's layer and is associated with mutations in the TGFBI gene (56). PTK is helpful for the treatment of this kind of dystrophy as this method has noninvasive nature and results in remarkable visual improvement (57). Nevertheless, there are few reports of effective treatment with SK in these patients. Schwartz and Hugh reported successful management of a 52-year-old female with Reis-Bucklers' dystrophy with SK (58). Additionally, McGrath et al. reported a series of 3 patients with Reis-Bucklers' dystrophy treated with SK. They utilized MMC as adjunctive treatment (28).

Climatic droplet keratopathy (CDK) is a degenerative corneal disorder characterized by the accumulation of translucent globules of different sizes located in the superficial corneal layers. Rao et al. reported a series of 15 patients with CDK who underwent SK for their severe symptoms or reduced visual acuity (29). During a follow-up period of 27 months, only 6% of them showed a return of symptoms. Vision improved in 12 out of 15 patients. For the remaining three patients, vision remained unchanged.

In conclusion, SK should be considered an effective and safe method for corneal dystrophies and degenerations located at the epithelial, basement membrane, Bowman's layer, and anterior stromal layers of the cornea. However, laser-based techniques such as PTK have advantages like more precise ablation depth and the regular plane of dissection (59).

### Bullous Keratopathy

Bullous keratopathy (BK) is the presence of bullous and corneal oedema caused by endothelial decompensation. Patients usually complain of blurring of vision or pain from the presence of bullae in the cornea. Surgical trauma is the leading cause of BK. Corneal transplantation is the modality of choice in a patient with BK and good visual potential (60). Other surgical options include bandage contact lens, ASP, PTK, collagen crosslinking and AMT (61). SK combined with AMT has been utilized to decrease haziness and alleviate discomfort in phakic patients with bullous keratopathy and poor visual prognosis (45), pseudophakic (62) and aphakic bullous keratopathy (12) (Table 2). AMT can serve as a bandage for a damaged cornea and act as a base for the epithelial cells to grow. Bae et al. reported cases with aphakic and pseudophakic bullous keratopathy managed by SK to improve visual acuity (VA) (12). Shalabi et al. proposed a combination of SK, cautery, and AMT to relieve symptoms more effectively in 4 cases of bullous keratopathy. After 16 months of follow-up, three patients remained symptomatic-free (45).

In summary, SK is only indicated for BK in patients with poor visual prognosis; however, it is shown to be effective in reducing symptoms and improving VA, especially in conjunction with AMT.

### Corneal Haziness

SK is an effective surgical technique to reduce corneal haziness. This method has been utilized to diminish haziness in corneal scarring, corneal neovascularization, keloid, and post photorefractive keratectomy (PRK) haziness. Fernandes et al. reported three eyes with post anterior stromal puncture subepithelial fibrosis, which was managed by SK. All patients' symptoms were alleviated, and at the final follow-up, there was no recurrence (46). Corneal haziness is a potential complication of PRK (63). Available options for managing this complication include conservative management with corticosteroids, PTK or SK. Khakshoor et al. conducted a study on the effectiveness of SK in reducing haziness, revealing that adjunctive MMC might improve the results (40). All nine patients who underwent SK+MMC experienced a clearer sight. None of the patients had a recurrence of haziness during the follow-up period.

Chronic inflammation sometimes leads to the formation of new vessels and pseudopterygium on the cornea. Qian et al. performed SK and administered an injection of subconjunctival Bevacizumab for treatment of the secondary superficial neovascularization (41). They reported regression of neovascularization following this procedure after 3 months of follow-up.

Although SK has been widely used to manage corneal scarring for various etiologies, little is reported in the literature (Table 2). Ozgurhan et al. reported four cases of corneal scarring and astigmatism post pterygium surgery that were treated by SK. McGrath also reported 8 cases of corneal scarring treated with SK plus MMC (28). These studies show SK is successful in the reduction of scarring and improvement of vision.

Corneal keloid is a rare disorder that is characterized by the proliferation of collagen tissue in the cornea in place of previous trauma or surgery. It can occur years after the initial trauma, as opposed to a hypertrophic scar (64). Surgeons have tried many different techniques to treat corneal keloids, including SK with or without PTK, PTK, lamellar keratectomy (LK), or penetrating keratoplasty (PK and implantation of keratoprosthesis) (38). Despite the method of surgery, reappearance is common. SK is one of the most common surgeries for visually significant keloids. Adjunctive use of AMT or MMC has been applied to lessen recurrence (65). Lee et al. reported 4 cases of keloid treated with SK (3 patients additionally received AMT). After 10 months of follow-up, all of them experienced a recurrence of symptoms (38). In addition, Bakhtiari et al. reported two cases of keloid who underwent SK; both required a second intervention (36). Nevertheless, none of three patients of corneal keloid treated with SK and AMT in the series reported by Gupta experienced recurrence (37). In cases of corneal keloid, PK is often complicated and results in graft failure; also, recurrence is common; therefore, SK should be considered a substitute.

A very rare indication for SK was reported by Chaurasia et al. (66). They reported a five-month-old boy with epidermolysis bullosa presented with the congenital whitish raised lesion. As the lesions were visually significant, the surgeon decided to perform SK with AMT. The surgery was successful, and the cornea became transparent and lesion-free during 4 years of observation.

### SK as an Adjunct to Other Surgical Procedures

Excision of pterygium is one of the most common ocular surgeries. To remove a pterygium, the surgeon removes the neck and body of the pterygium, and then residual tissues are scraped off by a surgical blade layer to produce a clear cornea. The second step in pterygium surgery (scraping pterygium's head) is very similar to SK, and few studies used the term “SK” for that step: Seid et al. reported performing SK deep to Bowman's in the excised area followed by a free conjunctival graft resulting in lower recurrence rate (67). Additionally, Narasimhaiah et al. compared motorized diamond burr polishing (DBSK) with manual SK after pterygium excision. They found DBSK resulted in better astigmatism correction and better UCVA (68). SK can also be used in corneal scarring and induced astigmatism after pterygium excision (39). Nevertheless, most ophthalmologists consider pterygium excision as a different technique, and therefore we did not include data from studies on pterygium excision in this review.

Performing amniotic membrane transplantation (AMT) for ocular surface diseases is extremely common nowadays. It has been widely adopted in the management of conjunctival disorders, symblepharon, persistent corneal epithelial defects (69), bullous keratopathy, partial or total limbal stem cell deficiency (LSCD), chemical burn and as an adjunct to other surgical procedures to decrease ocular surface inflammation (70). In patients with a corneal surface disorder related to limbal cell deficiency, the corneal lesion is peeled using SK, followed by AMT transplantation. Anderson et al. reported improved BCVA and abolition of pain and photophobia with this technique for LSCD of various etiologies (71).

### Other Indications

SK has been utilized for many other diagnostic and therapeutic reasons. Although uncommon, SK can be performed in suspected keratitis resistant to conventional antibiotic therapy by reducing the biofilm in place. There are reported cases of fungal keratitis [Dematiaceous (72), Acremonium (73)], infectious crystalline keratopathy (74), and Fuchs marginal keratitis (75), in which SK was helpful. Additionally, Agarwal et al. proposed the use of SK in patients with Mooren's ulcer resulted in lessened antigenic stimuli and, therefore, can be efficacious (76). SK can also be adopted to remove corneal foreign bodies if not deeply positioned in stromal layers.

Although topical chemotherapy is the first choice for ocular surface squamous neoplasms, epithelial debridement is also utilized for both diagnosis and treatment of corneal and conjunctival tumors spread to the cornea. There are also reports of using SK for these purposes. Arya et al. reported a case of an 80-year-old male presenting with a corneal mass. The lesion was excised by SK, and the histology revealed the lesion was squamous cell carcinoma (77). There are other case reports with similar scenarios. Arepalli et al. reported a series of 15 patients with conjunctival squamous cell carcinoma treated with the combination of SK, cryotherapy, and plaque radiation (78). Askolis et al. suggested AMT as an adjunct treatment for SK in reconstructing the epithelial defect following the debridement of larger tumors.

Deposition of immunoglobulin in the subepithelial layer of the cornea is a very rare entity. Patients usually present with photophobia and pain, and slit-lamp examination shows gray-white gelatinous deposits on the cornea. Lesions are usually removed by SK, and further histological evaluation reveals an immunological origin (79, 80).

Some authors also used SK to remove VKC plaque from the cornea. Pelgerin et al. reported a case of a nine-year-old boy with a corneal ulcer due to VKC, which was resistant to medical therapy. They performed SK to remove plaques and placed AMT on the cornea. An improved BCVA and accelerated epithelialization were achieved (81).

### Complications

Subepithelial haze is the most reported complication of SK. This complication is usually benign and resolves without scarring (19). In patients treated with SK due to various etiologies, the subepithelial haze was reported from 0 to 35 % and in the DBSK method ranged from 0 to 26% (Table 1). However, there is not enough data to support the superiority of each method on this matter, and further studies are required.

Recurrence is also frequently reported. The rate of recurrence depends on the corneal pathology (Table 2). Researchers proposed several methods to reduce the recurrence, such as chelation with EDTA (12) in the treatment of band keratopathy or application of MMC in the treatment of SND (23, 28, 82).

Corneal infiltration is rarely reported in the literature as a consequence SK (15). Spontaneous reactivation of herpetic keratitis is also reported by Aldave et al. (18).

### Outcomes

SK is a simple method for the management of corneal pathologies. Studies showed that this method is effective in the treatment of RCES, with a success rate of 76% to 100% for simple SK (4, 5, 12, 42) and 85.2% to 100 % for DBSK (13–17, 19–21, 43, 82). In addition, SK improved BCVA and reduced the corneal astigmatism of patients with SND with a success rate of 78% to 100% (12, 22–25, 44, 80). The success rate in patients with band keratopathy who managed with SK ranged from 72 to 100% (12, 27–29). These success rates are comparable to the result of PTK. A literature review by Nagpal et al. revealed that the success rate of PTK in patients with RCES, SND, and BK were 46–100%, 86–100%, and 40–100%, respectively (59). Further clinical trials are required to determine the best method for each etiology. This review showed that corneal dystrophies could also be managed with SK, with a success rate ranging from 75 to 100% (28, 29, 31–35). Furthermore, patients with corneal haziness due to various etiologies such as corneal scarring (28), post-PRK haziness (40), and bullous keratopathy (45) were successfully managed with SK. However, due to a paucity of data on the success rate of SK for these etiologies, well-designed clinical trials are necessary.

## Future Directions

SK with the aid of additional techniques such as MMC or diamond burr polishing may be more beneficial than simple SK with a blade in reducing the rate of recurrence for its various indications. However, there are no RCTs to demonstrate this hypothesis. Furthermore, for many of its indications, there is a paucity of data in terms of outcomes. In addition, most of the case series patients were only followed for a short period. Despite newer trending techniques such as excimer laser PTK, with an expanding portfolio of indications, SK should be considered an effective and feasible therapeutic option for several corneal disorders.

## Conclusion

Superficial keratectomy is a simple and effective modality that is used to treat ocular surface disorders. It is both a therapeutic and diagnostic technique. The most common indications for SK are reduced vision and recurrent corneal erosions due to pathology in the superficial layers of the cornea. Despite the recent emergence of laser-assisted techniques such as excimer-based phototherapeutic keratectomy, SK has remained clinically relevant. Whilst PTK achieves a clear and smooth plane of dissection and better determines the depth of dissection, SK has key advantages over PTK in the simplicity of technique and cost-effectiveness. This review of the literature also revealed that the postoperative results of SK, for some indications such as recurrent epithelial erosion, are either matched or superior to PTK.

## Author Contributions

MZ-G conceptualized the idea and scope of the review. FS and AB both performed a literature review. FS wrote the manuscript and created the figures. All authors critically revised and edited the manuscript.

## Conflict of Interest

The authors declare that the research was conducted in the absence of any commercial or financial relationships that could be construed as a potential conflict of interest.

## Publisher's Note

All claims expressed in this article are solely those of the authors and do not necessarily represent those of their affiliated organizations, or those of the publisher, the editors and the reviewers. Any product that may be evaluated in this article, or claim that may be made by its manufacturer, is not guaranteed or endorsed by the publisher.
