# Supplementary material for: Superficial Keratectomy: A Review of Literature
Source: Front Med (Lausanne). 2022 Jul 6;9:915284. doi: 10.3389/fmed.2022.915284 (PMC9299356; doi:10.3389/fmed.2022.915284)
Supplement: Supplementary file 1 [file Data_Sheet_1.docx]

Records identified through database searching
(n = 568)

**Screening**

**Included**

**Identification**

Additional records identified through hand searched
(n = 20)

Records after duplicates removed
(n = 568)

Records screened
(n = 139)

Case report
(n =39)

Case series:

(100)

Outcomes (35)

Indications or technique (65)
